# Diaqua­bis(2,2′-bi-1*H*-imidazole-κ^2^ *N* ^3^,*N* ^3′^)nickel(II) bis­(3-methyl­benzoate) 3-methyl­benzoic acid disolvate

**DOI:** 10.1107/S1600536809044705

**Published:** 2009-11-04

**Authors:** Zhou Hui

**Affiliations:** a College of Chemistry and Chemical Engineering, Henan University, Kaifeng 475001, People’s Republic of China

## Abstract

In the title compound, [Ni(C_6_H_6_N_4_)_2_(H_2_O)_2_](C_8_H_7_O_2_)_2_·2C_8_H_8_O_2_, the Ni^II^ atom (site symmetry 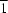) is coordinated by two *N*,*N*′-bidentate 2,2′-biimidazole ligands and two water mol­ecules, resulting in a slightly distorted *trans*-NiO_2_N_4_ geometry for the metal ion. In the crystal, the components are linked by N—H⋯O and O—H⋯O hydrogen bonds, generating an infinite two-dimensional network running parallel to (100). The methyl group of the benzoic acid mol­ecule is disordered over two sites in a 0.563 (17):0.437 (17) ratio.

## Related literature

For a related structure, see: Yang *et al.* (2009 ▶).
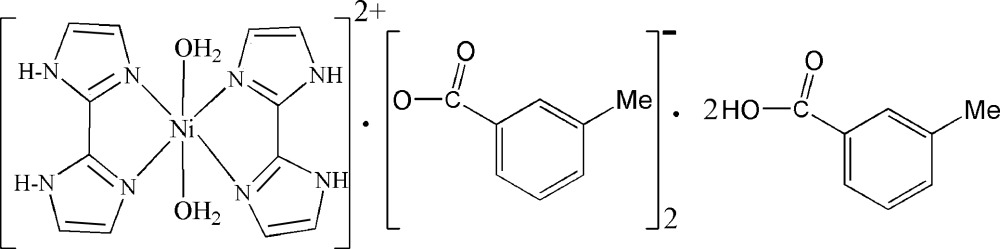


## Experimental

### 

#### Crystal data

[Ni(C_6_H_6_N_4_)_2_(H_2_O)_2_](C_8_H_7_O_2_)_2_·2C_8_H_8_O_2_
*M* *_r_* = 905.60
Monoclinic, 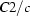

*a* = 34.747 (15) Å
*b* = 9.237 (4) Å
*c* = 14.099 (6) Å
β = 93.564 (8)°
*V* = 4516 (3) Å^3^
*Z* = 4
Mo *K*α radiation
μ = 0.50 mm^−1^
*T* = 296 K
0.25 × 0.19 × 0.13 mm

#### Data collection

Bruker SMART APEX CCD diffractometer
Absorption correction: multi-scan (*SADABS*; Bruker, 2001 ▶) *T*_min_ = 0.886, *T*_max_ = 0.939
12088 measured reflections
4417 independent reflections
2926 reflections with *I* > 2σ(*I*)
*R*_int_ = 0.057

#### Refinement

*R*[*F* ^2^ > 2σ(*F* ^2^)] = 0.091
*wR*(*F* ^2^) = 0.269
*S* = 1.00
4417 reflections
287 parameters
12 restraints
H atoms treated by a mixture of independent and constrained refinement
Δρ_max_ = 2.27 e Å^−3^
Δρ_min_ = −0.39 e Å^−3^

### 

Data collection: *SMART* (Bruker, 2001 ▶); cell refinement: *SAINT-Plus* (Bruker, 2001 ▶); data reduction: *SAINT-Plus*; program(s) used to solve structure: *SHELXS97* (Sheldrick, 2008 ▶); program(s) used to refine structure: *SHELXL97* (Sheldrick, 2008 ▶); molecular graphics: *PLATON* (Spek, 2009 ▶); software used to prepare material for publication: *PLATON*.

## Supplementary Material

Crystal structure: contains datablocks global, I. DOI: 10.1107/S1600536809044705/hb5141sup1.cif

Structure factors: contains datablocks I. DOI: 10.1107/S1600536809044705/hb5141Isup2.hkl

Additional supplementary materials:  crystallographic information; 3D view; checkCIF report

## Figures and Tables

**Table 1:** Selected bond lengths (Å)

Ni1—N1	2.092 (3)
Ni1—N4	2.097 (3)
Ni1—O1*W*	2.105 (3)

**Table 2:** Hydrogen-bond geometry (Å, °)

*D*—H⋯*A*	*D*—H	H⋯*A*	*D*⋯*A*	*D*—H⋯*A*
O4—H4⋯O2^i^	0.852 (8)	1.759 (9)	2.606 (4)	172 (3)
O1*W*—H1*WB*⋯O3	0.849 (7)	2.094 (10)	2.897 (4)	158 (2)
O1*W*—H1*WA*⋯O1	0.847 (7)	1.819 (8)	2.649 (3)	166 (2)
N3—H3*A*⋯O2^ii^	0.890 (9)	1.920 (12)	2.763 (3)	157 (2)
N2—H2*A*⋯O1^ii^	0.889 (8)	1.854 (9)	2.732 (3)	169.4 (19)

Symmetry codes: (i) 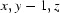; (ii) 
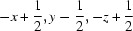
.

**Fig. 1.**
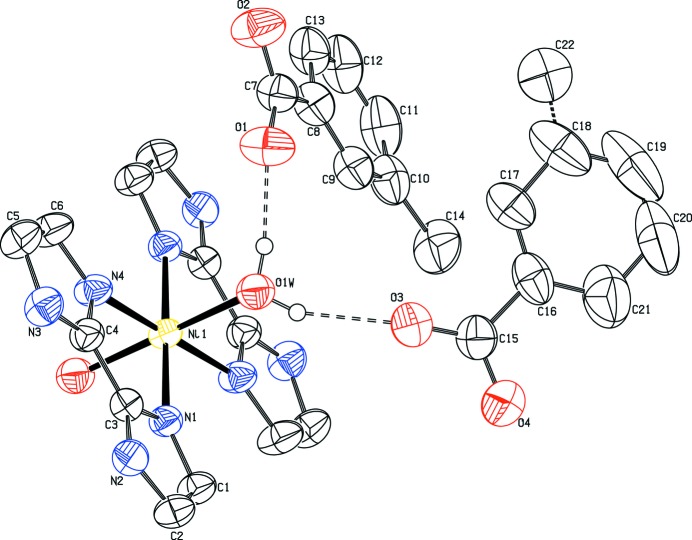
Molecular structure of (I), showing displacement ellipsoids drawn at the 30% probability level. H-bonds are shown as dashed lines. Disordered part of C22' and H atoms not involved in the hydrogen bonds have been omitted for the clarity. Unlabeled atoms are related to labeled atoms by the symmetry transformation 1/2 - x, 1/2 - y, -z.

**Fig. 2.**
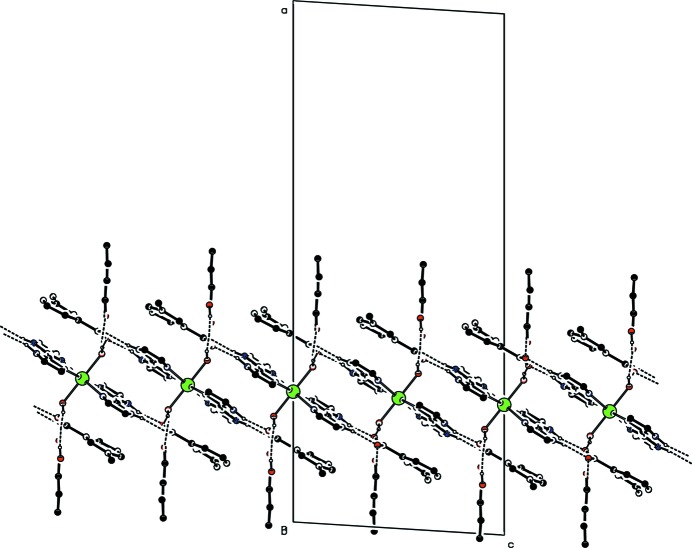
Part of the crystal structure of (I), showing the formation of the two-dimensional network along the (100) direction. Hydrogen bonds are shown as dashed lines. Disordered part of C22' and H atoms not involved in the hydrogen bonds have been omitted for the clarity.

## supplementary crystallographic information

### Comment

2,2'-Biimidazole is an interesting ligand because it has two N sites and two –NH donors. Both N-donors having the stronger coordination ability and flexible coordination modes. Moreover, two –NH donors can interact with other hydrogen bonding acceptors *via* hydrogen bonds (Yang *et al.*, 2009). Herein, we report the title compound, (I).

In the symmetric unit of (I), Ni^2+^ having an inversion centre is coordinated by two water molecules occupied the axial sites and two 2,2'-biimidazole ligands through respective two N atoms occupied the equatorial plane, which results in a more regular octahedron (Ni1—N1 2.092 (3) Å; Ni1—N4 2.097 (3) Å; Ni1—O1W 2.105 (3) Å). Each water molecule interacts with 3-methyl-benzenecarboxylate and 3-methyl-benzenecarboxylic acid through O1W—H1WA···O1 and O1W—H1WA···O3 hydrogen bonds (Fig. 1). Adjacent units are linked together by two pairs of N2—H2A···O1 and N3—H3A···O1, and one O4—H4···O2 hydrogen bonds into an infinite two-dimensional network along the (100) direction (Fig. 2).

### Experimental

NiSO_4_.6H_2_O(0.18 g, 0.70 mmol) was added into the aqueous solution (15 ml) including 3-methyl-benzenecarboxylic acid (0.14 g, 1.0 mmol) and NaOH (0.04 g, 1.0 mmol) and refluxed for 30 min. Then an ethanol solution (10 ml) containing 2,2'-biimidazole (0.08 g, 0.60 mmol) was slowly added with continuous stirring. The resulting solution was refluxed for 3 h, filtered and kept for crystallization. After nine days, green blocks of (I) were obtained.

### Refinement

H atoms bonded to N atoms, carboxyl and water O atoms are located from the difference maps and refined isotropically with 0.89 (1) Å for N—H, 0.85 (1) Å for O—H and the distance H···H = 1.34 (1) Å from water molecule using *DFIX* commands, respectively. All the remaining H atoms were positioned geometrically with C–H = 0.93 Å (aromatic) and 0.96 Å (methyl) and were refined as riding with *U*_iso_(H) = 1.2*U*_eq_(C) (aromatic) and 1.5*U*_eq_(C) (methyl). The disordered methyl carbon was devided into two parts C22 and C22' with the anisotrophic displacement parameters 0.102 and 0.117, respectively. H atoms bound to them are not added.

### Crystal data

**Table:**

[Ni(C_6_H_6_N_4_)_2_(H_2_O)_2_](C_8_H_7_O_2_)_2_·2C_8_H_8_O_2_	*F*(000) = 1896
*M**_r_* = 905.60	*D*_x_ = 1.332 Mg m^−^^3^
Monoclinic, *C*2/*c*	Mo *K*α radiation, λ = 0.71073 Å
Hall symbol: -C 2yc	Cell parameters from 2285 reflections
*a* = 34.747 (15) Å	θ = 2.3–22.7°
*b* = 9.237 (4) Å	µ = 0.50 mm^−^^1^
*c* = 14.099 (6) Å	*T* = 296 K
β = 93.564 (8)°	Block, green
*V* = 4516 (3) Å^3^	0.25 × 0.19 × 0.13 mm
*Z* = 4	

### Data collection

**Table:**

Bruker SMART APEX CCD diffractometer	4417 independent reflections
Radiation source: fine-focus sealed tube	2926 reflections with *I* > 2σ(*I*)
graphite	*R*_int_ = 0.057
0.3° wide ω exposures scans	θ_max_ = 26.0°, θ_min_ = 2.3°
Absorption correction: multi-scan (*SADABS*; Bruker, 2001)	*h* = −41→42
*T*_min_ = 0.886, *T*_max_ = 0.939	*k* = −11→10
12088 measured reflections	*l* = −12→17

### Refinement

**Table:**

Refinement on *F*^2^	Secondary atom site location: difference Fourier map
Least-squares matrix: full	Hydrogen site location: inferred from neighbouring sites
*R*[*F*^2^ > 2σ(*F*^2^)] = 0.091	H atoms treated by a mixture of independent and constrained refinement
*wR*(*F*^2^) = 0.269	*w* = 1/[σ^2^(*F*_o_^2^) + (0.1377*P*)^2^ + 26.2495*P*] where *P* = (*F*_o_^2^ + 2*F*_c_^2^)/3
*S* = 1.00	(Δ/σ)_max_ < 0.001
4417 reflections	Δρ_max_ = 2.27 e Å^−^^3^
287 parameters	Δρ_min_ = −0.39 e Å^−^^3^
12 restraints	Extinction correction: *SHELXL97* (Sheldrick, 2008), Fc^*^=kFc[1+0.001xFc^2^λ^3^/sin(2θ)]^-1/4^
Primary atom site location: structure-invariant direct methods	Extinction coefficient: 0.0013 (2)

### Special details

**Table:**

Geometry. All e.s.d.'s (except the e.s.d. in the dihedral angle between two l.s. planes) are estimated using the full covariance matrix. The cell e.s.d.'s are taken into account individually in the estimation of e.s.d.'s in distances, angles and torsion angles; correlations between e.s.d.'s in cell parameters are only used when they are defined by crystal symmetry. An approximate (isotropic) treatment of cell e.s.d.'s is used for estimating e.s.d.'s involving l.s. planes.
Refinement. Refinement of *F*^2^ against ALL reflections. The weighted *R*-factor *wR* and goodness of fit *S* are based on *F*^2^, conventional *R*-factors *R* are based on *F*, with *F* set to zero for negative *F*^2^. The threshold expression of *F*^2^ > σ(*F*^2^) is used only for calculating *R*-factors(gt) *etc*. and is not relevant to the choice of reflections for refinement. *R*-factors based on *F*^2^ are statistically about twice as large as those based on *F*, and *R*- factors based on ALL data will be even larger.

### Fractional atomic coordinates and isotropic or equivalent isotropic displacement parameters (Å^2^)

**Table:**

	*x*	*y*	*z*	*U*_iso_*/*U*_eq_	Occ. (<1)
Ni1	0.2500	0.2500	0.0000	0.03434 (15)	
N1	0.23426 (8)	0.0763 (3)	0.08464 (19)	0.0378 (7)	
N2	0.20745 (8)	0.0135 (3)	0.2167 (2)	0.0439 (8)	
H2A	0.1934 (3)	0.014 (4)	0.2673 (7)	0.052 (11)*	
N3	0.18763 (9)	0.3465 (3)	0.2311 (2)	0.0497 (8)	
H3A	0.1819 (7)	0.2986 (18)	0.2832 (8)	0.052 (4)*	
N4	0.21821 (9)	0.3628 (3)	0.0979 (2)	0.0428 (8)	
O3	0.37232 (9)	0.1134 (4)	0.1108 (3)	0.0818 (11)	
O4	0.40602 (10)	−0.0866 (4)	0.1032 (3)	0.0912 (13)	
H4	0.3851 (3)	−0.1337 (17)	0.108 (2)	0.040 (10)*	
O1W	0.29960 (7)	0.2675 (2)	0.09288 (19)	0.0485 (7)	
H1WA	0.3112 (4)	0.3481 (6)	0.094 (2)	0.024 (8)*	
H1WB	0.3173 (3)	0.2048 (8)	0.090 (3)	0.065 (13)*	
C1	0.23884 (11)	−0.0697 (4)	0.0987 (3)	0.0453 (10)	
H1	0.2515	−0.1321	0.0593	0.054*	
C2	0.22193 (11)	−0.1089 (4)	0.1793 (2)	0.0455 (10)	
H2	0.2206	−0.2019	0.2041	0.055*	
C3	0.21533 (10)	0.1215 (4)	0.1581 (2)	0.0370 (8)	
C4	0.20634 (10)	0.2754 (4)	0.1646 (3)	0.0406 (9)	
C5	0.18747 (12)	0.4897 (4)	0.2047 (3)	0.0561 (11)	
H5	0.1767	0.5661	0.2368	0.067*	
C6	0.20573 (12)	0.4988 (4)	0.1241 (3)	0.0563 (11)	
H6	0.2095	0.5839	0.0906	0.068*	
C15	0.40297 (13)	0.0524 (5)	0.1077 (3)	0.0600 (12)	
C16	0.44042 (13)	0.1300 (6)	0.1130 (3)	0.0694 (14)	
C17	0.44083 (15)	0.2790 (6)	0.1168 (3)	0.0726 (15)	
H17	0.4175	0.3279	0.1184	0.087*	
C18	0.47472 (17)	0.3594 (7)	0.1183 (4)	0.1015 (19)	
C19	0.5081 (2)	0.2839 (10)	0.1168 (6)	0.144 (3)	
H19	0.5312	0.3347	0.1164	0.173*	
C20	0.50918 (19)	0.1346 (10)	0.1159 (6)	0.138 (3)	
H20	0.5328	0.0868	0.1188	0.166*	
C21	0.47463 (5)	0.05430 (17)	0.11043 (11)	0.116 (3)	
H21	0.4748	−0.0461	0.1053	0.140*	
C22	0.47839 (5)	0.50541 (17)	0.12443 (11)	0.102 (5)*	0.437 (17)
C22'	0.46819 (5)	0.54041 (17)	0.12053 (11)	0.117 (4)*	0.563 (17)
O1	0.32801 (5)	0.53214 (17)	0.11606 (11)	0.0665 (9)	
O2	0.34258 (5)	0.76257 (17)	0.10071 (11)	0.0758 (10)	
C7	0.34256 (5)	0.63145 (17)	0.07347 (11)	0.0528 (11)	
C8	0.36146 (5)	0.59948 (17)	−0.01813 (11)	0.0536 (11)	
C9	0.37173 (5)	0.45833 (17)	−0.04007 (11)	0.0560 (11)	
H9	0.3662	0.3844	0.0017	0.067*	
C10	0.38992 (13)	0.4239 (6)	−0.1220 (3)	0.0663 (14)	
C11	0.39556 (13)	0.5355 (7)	−0.1846 (3)	0.0798 (17)	
H11	0.4069	0.5150	−0.2411	0.096*	
C12	0.38518 (13)	0.6747 (7)	−0.1668 (3)	0.0777 (15)	
H12	0.3887	0.7467	−0.2116	0.093*	
C13	0.36927 (13)	0.7085 (5)	−0.0815 (3)	0.0660 (13)	
H13	0.3639	0.8043	−0.0670	0.079*	
C14	0.40365 (16)	0.2721 (6)	−0.1383 (4)	0.0880 (17)	
H14A	0.4071	0.2582	−0.2047	0.132*	
H14B	0.3849	0.2044	−0.1179	0.132*	
H14C	0.4277	0.2566	−0.1027	0.132*	

### Atomic displacement parameters (Å^2^)

**Table:**

	*U*^11^	*U*^22^	*U*^33^	*U*^12^	*U*^13^	*U*^23^
Ni1	0.0416 (3)	0.0285 (3)	0.0337 (3)	0.0008 (3)	0.0086 (2)	0.0003 (2)
N1	0.0382 (15)	0.0335 (14)	0.0427 (15)	−0.0014 (12)	0.0097 (12)	−0.0007 (12)
N2	0.0453 (16)	0.0449 (16)	0.0422 (15)	−0.0005 (13)	0.0087 (13)	0.0048 (13)
N3	0.0542 (17)	0.0502 (16)	0.0464 (16)	0.0046 (14)	0.0160 (13)	0.0003 (14)
N4	0.0506 (16)	0.0339 (14)	0.0457 (15)	−0.0016 (13)	0.0165 (13)	−0.0025 (12)
O3	0.0574 (18)	0.069 (2)	0.120 (3)	−0.0004 (16)	0.0135 (18)	−0.0039 (19)
O4	0.070 (2)	0.069 (2)	0.135 (3)	0.0079 (18)	0.016 (2)	−0.008 (2)
O1W	0.0504 (14)	0.0431 (14)	0.0523 (14)	−0.0011 (12)	0.0068 (11)	0.0002 (11)
C1	0.054 (2)	0.0344 (17)	0.0485 (19)	0.0021 (16)	0.0121 (16)	−0.0034 (15)
C2	0.060 (2)	0.0359 (17)	0.0407 (18)	−0.0001 (16)	0.0068 (16)	0.0080 (15)
C3	0.0375 (17)	0.0382 (17)	0.0353 (16)	−0.0004 (14)	0.0028 (13)	0.0007 (14)
C4	0.0418 (18)	0.0376 (18)	0.0432 (18)	0.0012 (14)	0.0074 (14)	−0.0022 (14)
C5	0.067 (2)	0.045 (2)	0.058 (2)	0.0105 (19)	0.0118 (19)	−0.0050 (18)
C6	0.077 (3)	0.0348 (18)	0.059 (2)	0.0029 (18)	0.025 (2)	−0.0019 (17)
C15	0.058 (2)	0.068 (3)	0.055 (2)	0.001 (2)	0.0119 (19)	0.001 (2)
C16	0.050 (2)	0.105 (4)	0.054 (2)	−0.007 (2)	0.0080 (19)	−0.012 (2)
C17	0.068 (3)	0.092 (3)	0.057 (3)	−0.022 (3)	0.005 (2)	−0.006 (2)
C18	0.095 (4)	0.146 (5)	0.065 (3)	−0.057 (3)	0.020 (3)	−0.025 (3)
C19	0.076 (4)	0.233 (8)	0.126 (6)	−0.068 (5)	0.022 (4)	−0.042 (5)
C20	0.053 (3)	0.207 (8)	0.153 (7)	0.003 (5)	−0.001 (4)	−0.027 (7)
C21	0.077 (4)	0.145 (6)	0.128 (5)	0.009 (4)	0.010 (4)	−0.038 (5)
O1	0.0949 (19)	0.0607 (16)	0.0472 (14)	−0.0204 (15)	0.0307 (14)	−0.0076 (13)
O2	0.101 (2)	0.0515 (16)	0.0805 (19)	−0.0076 (16)	0.0458 (16)	−0.0024 (14)
C7	0.056 (2)	0.054 (2)	0.049 (2)	−0.0049 (19)	0.0106 (17)	−0.0028 (18)
C8	0.054 (2)	0.070 (2)	0.0381 (18)	−0.005 (2)	0.0124 (16)	0.0022 (18)
C9	0.052 (2)	0.068 (2)	0.048 (2)	−0.003 (2)	0.0063 (18)	−0.0046 (19)
C10	0.051 (2)	0.094 (3)	0.053 (2)	0.000 (2)	0.0013 (19)	−0.015 (2)
C11	0.057 (2)	0.135 (4)	0.049 (2)	0.009 (3)	0.0175 (19)	0.006 (3)
C12	0.060 (3)	0.118 (4)	0.057 (2)	0.003 (3)	0.018 (2)	0.027 (3)
C13	0.055 (2)	0.081 (3)	0.063 (2)	−0.001 (2)	0.015 (2)	0.020 (2)
C14	0.083 (3)	0.102 (4)	0.080 (3)	0.015 (3)	0.015 (3)	−0.038 (3)

### Geometric parameters (Å, °)

**Table:**

Ni1—N1^i^	2.092 (3)	C16—C21	1.381 (5)
Ni1—N1	2.092 (3)	C16—C17	1.378 (7)
Ni1—N4^i^	2.097 (3)	C17—C18	1.391 (7)
Ni1—N4	2.097 (3)	C17—H17	0.9300
Ni1—O1W^i^	2.105 (3)	C18—C19	1.354 (10)
Ni1—O1W	2.105 (3)	C18—C22	1.357 (7)
N1—C3	1.328 (4)	C18—C22'	1.688 (7)
N1—C1	1.371 (4)	C19—C20	1.380 (12)
N2—C3	1.335 (4)	C19—H19	0.9300
N2—C2	1.357 (5)	C20—C21	1.409 (8)
N2—H2A	0.889 (8)	C20—H20	0.9300
N3—C4	1.345 (5)	C21—H21	0.9300
N3—C5	1.374 (5)	O1—C7	1.2229
N3—H3A	0.890 (9)	O2—C7	1.2706
N4—C4	1.325 (4)	C7—C8	1.5138
N4—C6	1.387 (5)	C8—C13	1.384 (5)
O3—C15	1.208 (5)	C8—C9	1.3917
O4—C15	1.290 (6)	C9—C10	1.388 (5)
O4—H4	0.852 (8)	C9—H9	0.9300
O1W—H1WA	0.847 (7)	C10—C11	1.379 (7)
O1W—H1WB	0.849 (7)	C10—C14	1.503 (7)
C1—C2	1.362 (5)	C11—C12	1.362 (8)
C1—H1	0.9300	C11—H11	0.9300
C2—H2	0.9300	C12—C13	1.391 (7)
C3—C4	1.460 (5)	C12—H12	0.9300
C5—C6	1.338 (6)	C13—H13	0.9300
C5—H5	0.9300	C14—H14A	0.9600
C6—H6	0.9300	C14—H14B	0.9600
C15—C16	1.483 (6)	C14—H14C	0.9600
			
N1^i^—Ni1—N1	180.0	O3—C15—C16	123.0 (4)
N1^i^—Ni1—N4^i^	80.71 (11)	O4—C15—C16	114.2 (4)
N1—Ni1—N4^i^	99.29 (11)	C21—C16—C17	120.0 (4)
N1^i^—Ni1—N4	99.29 (11)	C21—C16—C15	120.5 (4)
N1—Ni1—N4	80.71 (11)	C17—C16—C15	119.5 (4)
N4^i^—Ni1—N4	180.0	C16—C17—C18	122.7 (5)
N1^i^—Ni1—O1W^i^	86.39 (10)	C16—C17—H17	118.6
N1—Ni1—O1W^i^	93.61 (10)	C18—C17—H17	118.6
N4^i^—Ni1—O1W^i^	89.82 (11)	C19—C18—C22	115.8 (5)
N4—Ni1—O1W^i^	90.18 (11)	C19—C18—C17	116.7 (7)
N1^i^—Ni1—O1W	93.61 (10)	C22—C18—C17	127.4 (5)
N1—Ni1—O1W	86.39 (10)	C19—C18—C22'	128.8 (5)
N4^i^—Ni1—O1W	90.18 (11)	C22—C18—C22'	13.21 (8)
N4—Ni1—O1W	89.82 (11)	C17—C18—C22'	114.5 (5)
O1W^i^—Ni1—O1W	180.0	C18—C19—C20	122.6 (7)
C3—N1—C1	104.8 (3)	C18—C19—H19	118.7
C3—N1—Ni1	111.3 (2)	C20—C19—H19	118.7
C1—N1—Ni1	143.6 (2)	C19—C20—C21	120.1 (6)
C3—N2—C2	106.7 (3)	C19—C20—H20	119.9
C3—N2—H2A	129 (2)	C21—C20—H20	119.9
C2—N2—H2A	124 (2)	C16—C21—C20	117.6 (4)
C4—N3—C5	106.0 (3)	C16—C21—H21	121.2
C4—N3—H3A	118.4 (13)	C20—C21—H21	121.2
C5—N3—H3A	134.8 (12)	O1—C7—O2	124.0
C4—N4—C6	104.3 (3)	O1—C7—C8	119.1
C4—N4—Ni1	111.4 (2)	O2—C7—C8	116.9
C6—N4—Ni1	144.3 (2)	C13—C8—C9	118.4 (2)
C15—O4—H4	115.6 (12)	C13—C8—C7	121.4 (2)
Ni1—O1W—H1WA	116.6 (14)	C9—C8—C7	120.2
Ni1—O1W—H1WB	119 (2)	C10—C9—C8	122.4 (2)
H1WA—O1W—H1WB	104.7 (10)	C10—C9—H9	118.8
C2—C1—N1	109.3 (3)	C8—C9—H9	118.8
C2—C1—H1	125.4	C11—C10—C9	116.8 (4)
N1—C1—H1	125.4	C11—C10—C14	122.7 (4)
N2—C2—C1	107.0 (3)	C9—C10—C14	120.4 (4)
N2—C2—H2	126.5	C12—C11—C10	122.6 (4)
C1—C2—H2	126.5	C12—C11—H11	118.7
N1—C3—N2	112.3 (3)	C10—C11—H11	118.7
N1—C3—C4	118.2 (3)	C11—C12—C13	119.6 (5)
N2—C3—C4	129.5 (3)	C11—C12—H12	120.2
N4—C4—N3	112.5 (3)	C13—C12—H12	120.2
N4—C4—C3	118.1 (3)	C8—C13—C12	120.0 (4)
N3—C4—C3	129.4 (3)	C8—C13—H13	120.0
C6—C5—N3	107.3 (3)	C12—C13—H13	120.0
C6—C5—H5	126.4	C10—C14—H14A	109.5
N3—C5—H5	126.4	C10—C14—H14B	109.5
C5—C6—N4	110.0 (3)	H14A—C14—H14B	109.5
C5—C6—H6	125.0	C10—C14—H14C	109.5
N4—C6—H6	125.0	H14A—C14—H14C	109.5
O3—C15—O4	122.8 (4)	H14B—C14—H14C	109.5

Symmetry codes: (i) −*x*+1/2, −*y*+1/2, −*z*.

### Hydrogen-bond geometry (Å, °)

**Table:**

*D*—H···*A*	*D*—H	H···*A*	*D*···*A*	*D*—H···*A*
O4—H4···O2^ii^	0.85 (1)	1.76 (1)	2.606 (4)	172 (3)
O1W—H1WB···O3	0.85 (1)	2.09 (1)	2.897 (4)	158 (2)
O1W—H1WA···O1	0.85 (1)	1.82 (1)	2.649 (3)	166 (2)
N3—H3A···O2^iii^	0.89 (1)	1.92 (1)	2.763 (3)	157 (2)
N2—H2A···O1^iii^	0.89 (1)	1.85 (1)	2.732 (3)	169 (2)

Symmetry codes: (ii) *x*, *y*−1, *z*; (iii) −*x*+1/2, *y*−1/2, −*z*+1/2.
